# Clinical translation of TLR agonist-modified silicified cancer cell therapy supported by humanized mouse models

**DOI:** 10.1016/j.omton.2025.201074

**Published:** 2025-10-24

**Authors:** Mara P. Steinkamp, Danielle Burke, Madigan Morrison, Irina Lagutina, Lillian Fitzpatrick, Rita E. Serda

**Affiliations:** 1 Department of Pathology, University of New Mexico Health Science Center, Albuquerque, NM 87131, USA; 2 Internal Medicine, University of New Mexico Health Science Center, Albuquerque, NM 87131, USA; 3 University of New Mexico Comprehensive Cancer Center Animal Models Shared Resource, Albuquerque, NM 87131, USA

**Keywords:** silicified cancer cells, Toll-like receptor agonist, ovarian cancer, humanized mice, patient-derived xenograft, NBSGW, omentum, milky spots, cell-based therapy

## Abstract

Humanized patient-derived xenograft (huPDX) mouse models are crucial for evaluating the clinical translation of new immune therapeutics. Silicification of cancer cells renders the cells non-viable with enhanced stability and silica surface functionalization, enabling adsorption of pathogen-associated molecular patterns to attract and activate myeloid cells. While strong therapeutic efficacy has been demonstrated in syngeneic mouse models of ovarian cancer, promising results do not always translate to effective treatments for patients. To support clinical translation, therapeutic responses to silicified ovarian cancer cells were studied in huPDXs. Three humanized mouse models using different immunocompromised strains were used to evaluate recent advances in the field, including the addition of the *HHD* transgene and a *c-Kit* mutation to improve T cell activation and overall engraftment, respectively. In the final model, NBSGW-HLA-A2/HHD, intraperitoneal administration of silicified cancer cells reduced the tumor burden (*p* = 0.0006) in the presence of elevated T cells (*p* = 0.0001), indicating the activation of a robust anti-tumor immune response. Improvements in outcomes, including stable humanization and demonstration of effective immune therapy with Toll-like receptor (TLR)-agonist-modified silicified cancer cells, highlight improvements in humanized mouse models and support the translation of silicified cancer cell technology for women with ovarian cancer.

## Graphical abstract

**Figure:**
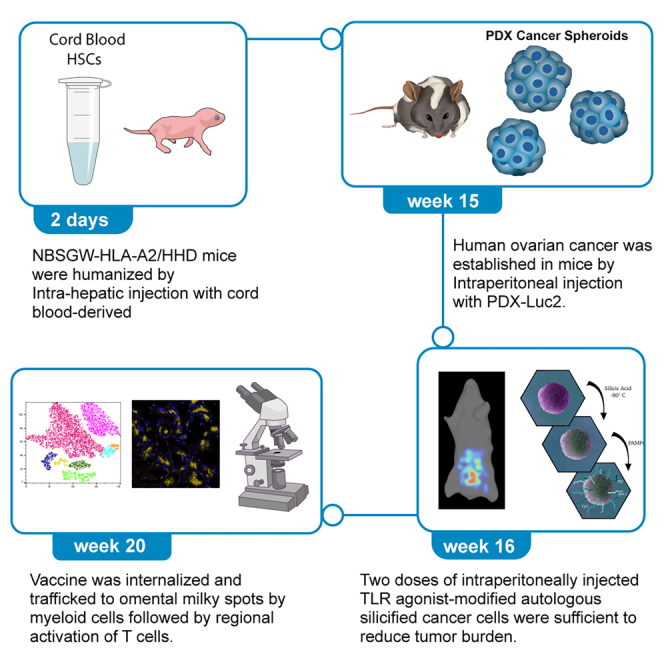

## Teaser

NBSGW mice crossed with HLA-A2/HHD mice have a *c-kit* mutation and human HLA class I heavy and light chains that limit the ability of mouse HSCs to compete with human HSCs and enable T cell selection against human antigens, enabling silicified cancer cells to activate human immune cells against cancer.

## Introduction

As cancer progresses, tumor cells recruit immunosuppressive immune cells and adapt to evade cancer-specific immune responses. Therapies that are able to effectively reactivate an anti-tumor response could revolutionize ovarian cancer treatment. However, current immunotherapies that target T cells with immune checkpoint blockade are effective in less than 10% of patients with ovarian cancer, even when combined with chemotherapy.^1^ Therefore, novel treatments are needed to improve immune activation. We have created a cell biomineralization process based on silicification to transform tumor cells into immune-activating entities that are readily recognized by and activate immune cells.^2^^,^^3^ Since cancer cells are highly heterogeneous from patient to patient, using a patient’s own tumor cells creates a personalized therapy that is customized to that patient’s cancer. Cell silicification renders tumor cells non-viable while preserving the cell’s integrity and its tumor antigens. Surface functionalization with Toll-like receptor (TLR) agonists (such as CpG 2006 oligonucleotide [ODN] and monophosphoryl lipid A [MPL]) enables the modified cancer cells to engage receptors on the surface of and, following phagocytosis, within antigen-presenting cells (APCs). Activation of TLRs stimulates increased expression of co-stimulatory molecules, enhances antigen presentation in the context of the major histocompatibility complex (MHC), and stimulates production of type I interferons and pro-inflammatory mediators.^4^^,^^5^^,^^6^ Treatment with TLR-functionalized silicified cancer cells facilitates the reversal of immune suppression in the tumor microenvironment, enabling activation and expansion of tumor-specific T cells.^2^^,^^3^ While our silicified cancer vaccines have demonstrated strong efficacy in syngeneic mouse models of ovarian and colorectal cancer, not all therapies tested in syngeneic mice translate to effective treatments in patients. Here, we have evaluated human immune responses to silicified cancer vaccine treatment using humanized patient-derived xenograft (huPDX) mouse models of ovarian cancer.

Our previous preclinical studies have shown that, following intraperitoneal (i.p.) injection in mice with ovarian cancer, fluorescently labeled TLR-agonist-modified silicified cancer cells are rapidly phagocytosed by ascites myeloid cells that then traffic the vaccine to the omentum and other adipose tissues in the peritoneal cavity.^3^ Within adipose tissues, vaccine-laden myeloid cells localize within fat-associated lymphoid clusters (FALCs),^3^ aggregates of leukocytes known to promote anti-cancer immune responses.^7^ The omentum is the primary site of ovarian cancer metastasis and contains FALCs, known as milky spots, with characteristics of both secondary lymphoid organs and tertiary lymphoid structures.^8^^,^^9^ Milky spots are aggregates of leukocytes located beneath the mesothelial cell layer of the omentum.^10^ Monocytes/macrophages comprise 70% of the leukocytes in human milky spots.^11^ Localization of myeloid cells laden with silicified cancer cell immune therapy in milky spots and other FALCs^3^ ideally positions them to promote anti-cancer immune responses. This is similar to findings by Christian et al., who reported that *Toxoplasma gondii* (parasite)-infected peritoneal macrophages in milky spots were responsible for priming CD8^+^ T cells, while conventional dendritic cells (cDC1s) provide secondary signals supporting T cell expansion and memory formation.^12^

For these studies, we have leveraged malignant ascites as an alternative source of ovarian cancer cells to develop personalized silicified cancer vaccines. Approximately 75% of patients with ovarian cancer present with disseminated disease at the time of diagnosis, and one-third of those patients have malignant ascites.^13^ We have established a PDX from ovarian cancer cells isolated from malignant ascites fluid. The ovarian cancer PDX established from human ascites retains the genetic heterogeneity of the primary tumor.^14^ Recently, we demonstrated that ovarian cancer PDX models could be established in humanized NBSGW (NOD.Cg-*Kit*^*W-41J*^*Tyr*^+^*Prkdc*^*scid*^*Il2rg*^*tm1Wjl*^/ThomJ) (huNBSGW) mice engrafted with human cord-blood-derived CD34^+^ hematopoietic stem cells (HSCs).^15^ The NBSGW strain is an immunocompromised strain on the NSG background carrying a *c-Kit* mutation that reduces the ability of mouse HSCs to compete with engrafted human HSCs. This allows for efficient humanization without the need for pre-engraftment irradiation, allowing early engraftment of human HSCs without exposing young mice to irradiation. Importantly, CD33^+^ myeloid progenitor cells are found in the bone marrow of huNBSGW, with myeloid cells making up greater than 5% of the human peripheral blood mononuclear cells in huNBSGW mice.^15^^,^^16^^,^^17^ The use of newer mouse strains for HSC engraftment has further improved human immune responses in humanized mice. The HLA-A2/HHD strain on the NSG background (NOD.Cg-*Prkdc*^*scid*^*Il2rg*^*tm1Wjl*^Tg(HLA-A/H2-D/B2M)1Dvs/SzJ) carries a transgene that expresses a human allele of the HLA class I heavy and light chains.^18^ Engrafting HLA-A-matched HSCs into the HLA-A2/HHD strain allows for T cell education in the mouse thymus and HLA-specific T cell responses in humanized mice. By breeding the NBSGW strain with the HLA-A2/HHD strain, we have established an immunocompromised model that can be engrafted with HLA-A-matched donor CD34^+^ HSCs as neonates so that the human T cell progenitors can be educated in the mouse thymus.

The PDX model PDX3 used in these studies was established from the malignant ascites of a patient with high-grade serous ovarian cancer. When engrafted into humanized mice, this PDX has been shown to increase the level of CD11b^+^ myeloid cells within the peritoneal cavity and recruit human macrophages and T cells into the tumor microenvironment.^19^ Because the silicified cancer cell vaccine requires both APCs and functional T cells, we chose to engraft PDX3 into humanized mice expressing a human MHC class 1 allele to allow for proper T cell maturation and HLA-specific T cell responses.

In this study, engrafting HLA-A-matched ovarian cancer PDX cells into huNBSGW-HLA-A2/HHD mice enabled us to evaluate the impact of i.p. vaccination with matched silicified cancer cells on regional immune cells and tumor progression. Transferrin- and folate-specific imaging agents were evaluated as noninvasive indicators of tumor burden.

## Results

### Silicified cancer cell immune therapy efficacy in humanized mouse models of ovarian cancer: NSG-A2 mice

**Figure 1:**
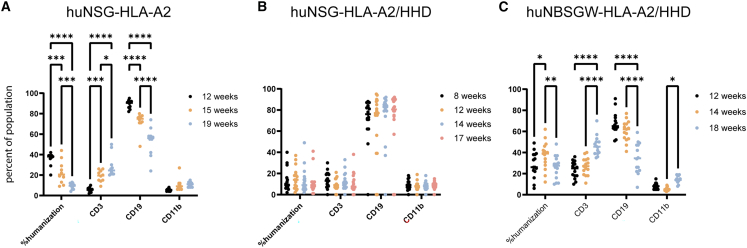
Immune cell composition and percentage of humanization by mouse model Flow cytometry was used to determine the percentage of humanization (human CD45^+^/total), T cell (CD3^+^), B cell (CD19), and myeloid cell (CD11b^+^) populations in peripheral blood from mice at various stages post-engraftment in three humanized mouse models: humanized NSG-HLA-A2 (A), NSG-HLA-A2/HHD (B), and NBSGW-HLA-A2/HHD (C) (Tukey's multiple comparison's test; ∗ *p* < 0.05, ∗∗ *p* < 0.01, ∗∗∗ *p* < 0.001, ∗∗∗∗ *p* < 0.0001).

**Figure 2:**
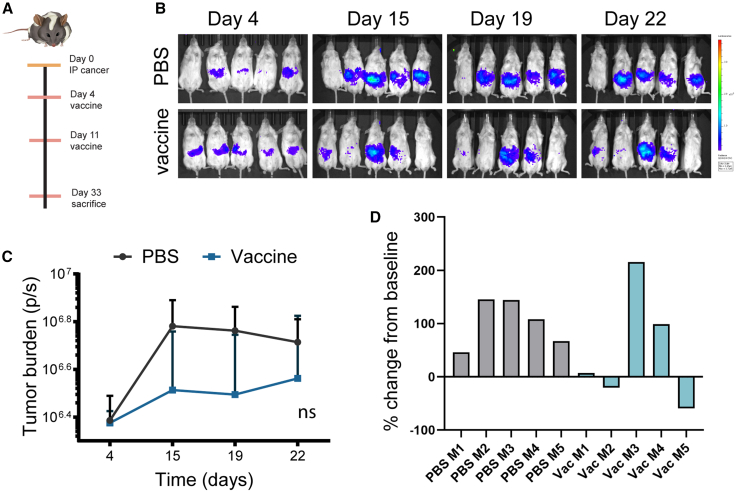
NSG humanized mice display an early therapeutic response to vaccination (A) Timeline. (B) Bioluminescent images of PDX3-Luc2-engrafted NSG-A2 humanized mice treated with vaccine or PBS across time. (C) Graph of tumor burden over time by treatment group. (D) Waterfall plot showing change in tumor burden on day 22 compared to day 4 as the baseline.

In support of clinical translation of silicified cancer cell vaccines, vaccine efficacy in humanized PDX mouse models of ovarian cancer was studied. Humanized mice were first established in 4-week-old NSG HLA-A2 mice expressing the human class 1 MHC HLA-A2.1 allele. Mice were irradiated with 90 centigray (cGy) followed by retro-orbital injection with 5 × 10^4^ HLA-matched (HLA-A2.1) cord-blood-derived CD34^+^ HSCs. At 12, 15, and 19 weeks post-HSC engraftment, mice were evaluated for immune cell humanization by flow cytometry using human- and mouse-specific antibodies (Figure 1A). Figures 1B and 1C present temporal humanization data for the humanized mouse models described in the succeeding sections but are shown here to compare outcomes from the three humanized mouse models evaluated. Mice with at least 20% of human CD45^+^ cells at 12 weeks were used in our study. By 15 weeks, the average humanization had dropped from 35% to 21% across NSG HLA-A2 groups (*p* < 0.001). This decrease in humanization may be caused by competition between mouse and human HSCs within the bone marrow niche. Average T cells increased from 5% to 19%, while myeloid cells increased from 6% to 11%. At 15 weeks post-HSC engraftment, disseminated peritoneal ovarian cancer was initiated by i.p. administration of 5 × 10^6^ PDX3-firefly2 luciferase (Luc2) (HLA-A2.1) cancer cells (day 0; *n* = 5). The tumor burden was monitored by bioluminescent imaging (luciferase expression). Mice were administered vaccine or phosphate-buffered saline (PBS) control on days 4 and 11 (Figure 2A), with the tumor burden shown across time in bioluminescent images (Figure 2B) or graphically (Figure 2C). Note that one mouse in the control group failed to develop tumors and was thus omitted from analysis. The remaining four PBS-group mice maintained an elevated tumor burden throughout the study, with the change in tumor burden from days 4 to 22 shown in a waterfall plot (Figure 2D). In this model, the percentage of humanization as measured in peripheral blood decreased over time. However, within the remaining engrafted human immune cells, the proportion of CD3^+^ T cells increased in the majority of mice, regardless of vaccination (Figure 1). In spite of the late engraftment of human cells and the low percentage of humanization, three of five vaccinated mice displayed a robust response to vaccination, with little or no tumor progression at day 22 (Figure 2D). Stochastic differences in tumor growth and response to treatment are seen across animal studies, particularly when using humanized mice that have variable percentages of humanization and differential maturation of immune cell subpopulations.^20^

**Figure 3:**
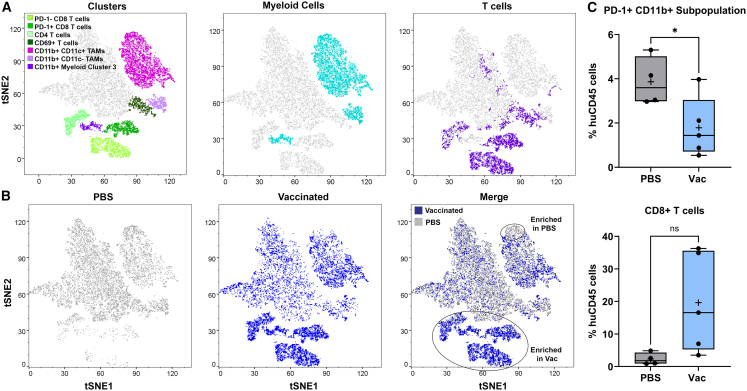
Vaccination of humanized NSG mice leads to an increase in human CD8^+^ cells and a decrease in suppressive myeloid cells in humanized mice with ovarian cancer (A) Myeloid and T cell t-SNE profiles. (B) Peritoneal immune cell populations in PBS- (*n* = 4) and vaccine- (*n* = 5) treated mice on day 33. (C) CD8^+^ T cells and PD-1^+^/CD11b^+^ myeloid cells present at day 33 by treatment group (unpaired *t* test with equal SD; ∗ *p* < 0.05).

Necropsy was performed on day 33, and cells in the tumor microenvironment (i.e., ascites) were characterized using spectral flow cytometry. t-SNE (t-distributed stochastic neighbor embedding) plots (plots showing spatial alignment of cells with common traits following statistical analysis of high-dimensional flow cytometry data) were used to identify three clusters of myeloid cells (CD11b^+^) and four clusters of T cells (CD3^+^) (Figure 3A). Treated and PBS control groups showed distinct immune cell populations (Figure 3B). Three of five vaccinated mice demonstrated elevated CD8^+^ T cells, greater than 7-fold higher compared to the PBS control (Figures 3B and 3C). This is consistent with murine cancer/vaccine studies, which show that vaccines stimulate a T cell-mediated immune response.^3^ The two vaccinated mice that did not show an increase in CD8^+^ T cells also had fewer total human immune cells in the ascites fluid. Significant differences between vaccine-treated and PBS control were also seen in the programmed cell death protein 1 (PD-1)^+^ CD11b^+^ myeloid population that is reduced by half in the vaccine-treated group (Figure 3C, *p* = 0.0389). While this is a small subpopulation of myeloid cells, it may be an indication that vaccine treatment promotes macrophage activation.

**Figure 4:**
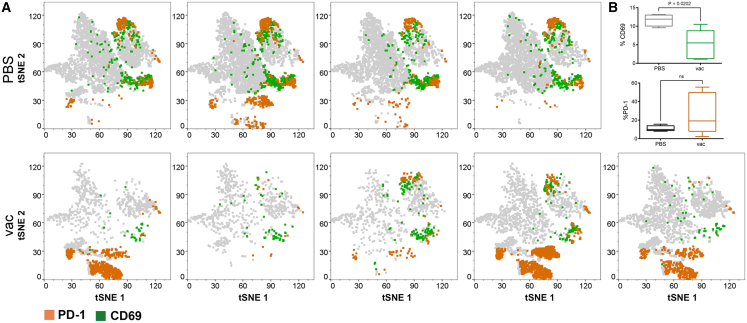
Vaccination of humanized NSG mice is associated with a reduction in CD69 expression by T cells (A) t-SNE analysis of CD69 and PD-1 expression in CD45^+^ cells on day 33 in PBS- or vaccine- (vac) treated mice. (B) Box and whisker plots showing CD69 and PD-1 expression at day 33 by treatment group (unpaired *t* test with equal SD).

CD69 is a membrane-bound receptor expressed by activated lymphocytes.^21^ It is considered a marker of tissue retention, with frequent expression by resident memory T cells in the tumor microenvironment.^22^ T cells isolated from tumor tissues have been shown to express more CD69 than circulating or non-tumor tissue T cells.^23^ In this study, CD69 was significantly elevated in unvaccinated, tumor-burdened mice compared to those treated with the vaccine (Figures 4A and 4B, *p* = 0.0202). While PD-1 was elevated in 3 of the 5 vaccinated mice, indicating that immune exhaustion was partly responsible for reducing vaccine efficacy at day 33, there was no significant change when comparing the two groups (Figure 4B). Interestingly, elevated PD-1 did not correlate with a high tumor burden in individual mice.

### NSG-HLA-A2/HHD mice

To improve human T cell responses, we bred NSG-HLA-A2/HHD mice in house to allow for neonatal engraftment of human HSCs. In addition, the HHD designation signifies the presence of HLA class I heavy and light chains, enabling T cell selection on a human transgene MHC. Cord-blood-derived CD34^+^ HSCs were engrafted by hepatic injection into 2-day-old NSG-HLA-A2/HHD neonates after pre-engraftment irradiation (70 cGy). The percentage of humanization was less than 20% in the majority of mice (Figure 1B). Mice were injected with PDX3-Luc2 (HLA-A2.1) ovarian cancer cells at week 14 (day 0). Half of the mice (*n* = 3) were vaccinated on days 4 and 11, and the rest (*n* = 4) were sham injected with PBS. All mice were euthanized on day 26, when the untreated controls showed abdominal distention.

Due to the loss of luciferase expression by the injected PDX3-Luc2 cancer cells, tumor burden was assessed using near-infrared (NIR) fluorescent probes. On day 26 post-tumor challenge, folate-receptor- and transferrin-receptor-targeted imaging agents were administered to the mice and evaluated as indicators of tumor burden. Mice were imaged live and at necropsy. The impact of the delivery route on imaging agent biodistribution was first investigated. Regardless of the route of administration (i.p. or intravenous [i.v.]), the IVISense Folate Receptor 680 probe predominantly accumulated in the kidneys in tumor-burdened mice. When the kidneys were removed from tissue analysis, tumor-specific fluorescence was detected with i.v. and i.p. delivery of IVISense Folate Receptor 680. Following i.v. administration, the majority of the signal was detected in tumor-burdened tissue (omentum, mesentery, ovaries, and fat pads), with little signal in the liver and spleen. Following i.p. administration, the signal was less tumor specific, with an equal intensity of signal in tumor-burdened tissues, liver, and spleen. Thus, i.v. administration was superior to i.p. injection for folate receptor imaging. The transferrin receptor probe localized primarily to the liver when administered i.v., with the signal also detected in the tumor-burdened ovary. Conversely, following i.p. administration, the highest signal for the IVISense Transferrin Receptor 750 probe was in tumor-burdened tissue (fat pads, omentum, and mesentery), making i.p. delivery superior for the transferrin receptor probe.

**Figure 5:**
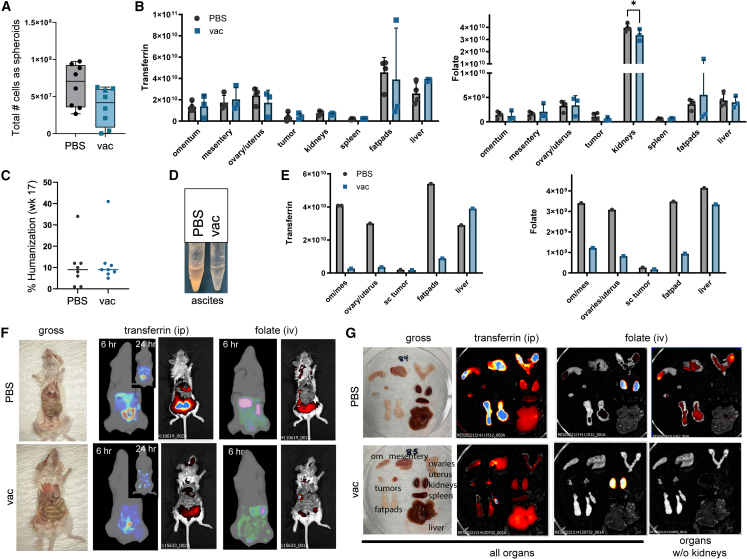
Folate receptor 680 and transferrin receptor 750 IVISense biodistribution in vaccinated or control tumor-bearing mice PDX3 ovarian tumor-bearing NSG-2A/HHD female mice and harvested organs were imaged using the IVIS Spectrum 24 h post-administration of IVISense Folate Receptor 680 (i.v.) or Transferrin Receptor 750 (i.p.). (A) Number of cancer cells as spheroids present in the ascites fluid by treatment group (not significant [ns]). (B) Tissue biodistribution presented as whole-organ (ROI) photons by treatment group and imaging agent (unpaired *t* test with equal SD; ∗ *p* < 0.05). (C) Mouse percentage of humanization. (D) Ascites from mice with high humanization by treatment group. (E) Imaging agent biodistribution in the control and vaccinated mice with the highest humanization. (F and G) Gross and fluorescent images of mice (F) and excised organs (G).

In our NSG-HLA-A2/HHD vaccine study, vaccinated mice had a median total number of spheroids that was less than that of the control group (Figure 5A, non-significant), indicating that the vaccine may have had an effect on tumor burden. However, the differences between the groups were not significant. Quantitation of fluorescent images of mice at 24 h post-injection showed specific localization of fluorescent IVISense Transferrin Receptor 750 and IVISense Folate Receptor 680 in the peritoneal cavity. Differences in tumor burden between untreated and vaccinated mice were not significant for these mice with low (10%–12%) humanization (Figure 5B).

However, two mice (one vaccinated: 41% human CD45 and one untreated: 34% human CD45) had similarly high humanization (Figure 5C). When comparing these two tumor-bearing mice, clear differences were seen. The ascites fluid was transparent in the vaccinated mouse and highly opaque in the untreated mouse (Figure 5D). The ascites fluid of the vaccinated mouse had no cancer spheroids present (total cell count [singlets]: 3.0 × 10^5^), while the PDX control ascites fluid contained many cancer spheroids (total cell count: 3.8 × 10^7^). Fluorescent transferrin receptor probe and folate receptor probe localization 24 h post-imaging agent injection in the two mice supported high probe accumulation in tumor-bearing tissues (fat pads, omentum, ovary, and mesentery) of the untreated mouse (Figures 5E–5G). Very little tumor-specific signal was seen in the vaccinated mouse. While the reduction in tumor burden in the highly humanized mouse is promising, no conclusions can be drawn until future studies are performed in a larger group of mice with adequate humanization.

### NBSGW-A2-HHD mice

**Figure 6:**
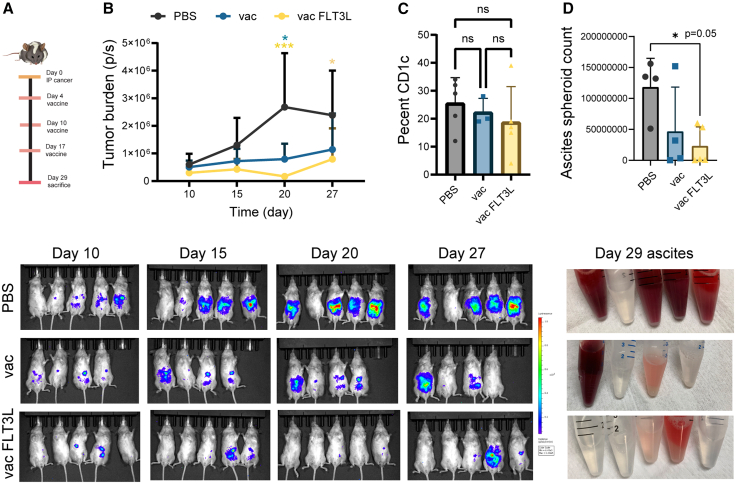
Vaccination of humanized NBSGW-HLA-A2/HHD mice reduces tumor burden (A) Treatment timeline. (B) Tumor burden and bioluminescent images of NBSGW-HLA-A2/HHD mice challenged with PDX3-Luc2 ovarian cancer by treatment group over time. Mice received PBS control, vaccine, or vaccine plus FLT3-L. (C) Percentage of CD1c^+^ human CD45^+^ cells in blood on day 29. (D) Cancer spheroid counts in ascites collected from mice on day 29 by treatment group. Images below the graph show conical tubes with ascites for each mouse by group. Graphs show means plus SD with unpaired two-tailed *t* test comparisons (∗ *p* < 0.05, ∗∗∗ *p* < 0.001).

To improve HSC engraftment in NSG-HLA-A2/HHD neonates, we bred HLA-A2/HHD onto the NBSGW background. NBSGW mice have a mutation in the *c-Kit* gene and do not require pre-engraftment irradiation, which can stunt the growth of neonates. The resulting NBSGW-HLA-A2/HHD mice showed large improvements in engraftment compared to the NSG-HLA-A2/HHD model (Figure 1C). 2-day-old neonates were injected via temporal vein with 4 × 10^4^ HLA-A2.1-matched cord-blood-derived CD34^+^ HSCs.^24^ At 12 weeks post-engraftment, the average humanization was 28%, and it increased to 37% at 14 weeks (Figure 1). During this time, T cells increased from 23% to 26%, and myeloid cells decreased from 8% to 5%. At 15 weeks of age, mice were challenged with PDX3-Luc2 (HLA-A2.1) tumor cells (day 0). The tumor burden was monitored using bioluminescence (luciferase expression) (Figure 6A). All mice in the PBS (control; *n* = 5) group showed tumor progression, while mice in the vaccinated group (*n* = 4, days 4, 10, and 17; Figure 6A) displayed an arrest of tumor growth (Figure 6B, *p* = 0.018, day 20). Another vaccinated group was treated with the hematopoietic cytokine FMS-like tyrosine kinase 3 ligand (FLT3-L) to support differentiation and survival of DCs.^25^ FLT3-L treatment did not appear to stably enhance the CD1c^+^ DC population, based on an analysis of blood from mice on day 29 (Figure 6C). This population of human DCs was of interest based on the expression of TLR4, while the CD141^+^ cDC population lacks expression of both TLR4 and TLR9.^25^ However, the FLT3-L-treated group had nearly undetectable bioluminescence at day 20, indicating a robust response to vaccination (p = 0.0006 [day 20] and p = 0.0370 [day 27] compared to PBS control). One explanation could be that the FLT3-L led to a transient increase in APC populations that boosted vaccine response. Consistent with the bioluminescence data, ascites collected on day 29 from control mice contained abundant cancer spheroids, while eight of nine mice in the two vaccinated groups had fewer cancer spheroids and significantly lower cell counts (Figure 6D). In fact, five of nine mice in the vaccinated groups had clear ascites fluid, no visible spheroids, and very low cell counts.

**Figure 7:**
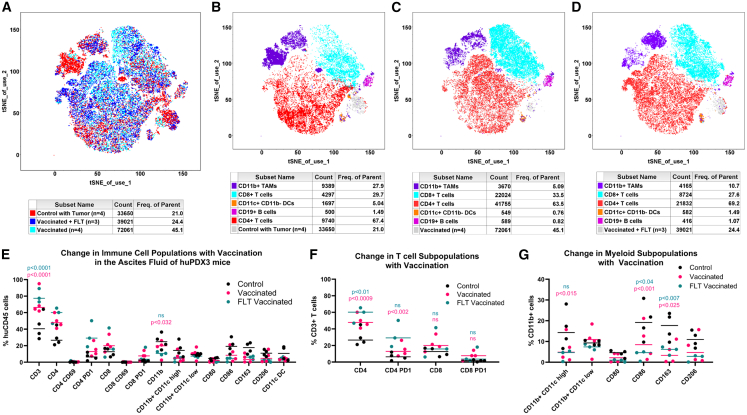
Vaccination of huNBSGW-HLA-A2/HHD mice with ovarian cancer reduces CD206^+^ TAMs and elevates CD4^+^ T cells (A) t-SNE plot of all mice color coded by group: control (red), vaccinated (cyan), and vaccinated plus FLT3-L (dark blue). (B–D) Clustering of immune cell subpopulations in control mice (B), vaccinated mice (C), and vaccinated FLT3-L-treated mice (D). (E) Percentage of each cell type within the huCD45^+^ population. (F) Analysis of T cell subpopulations. (G) Analysis of CD11b^+^ myeloid populations. Unpaired two-tailed *t* test comparisons were used to determine *p* values.

29 days post-tumor injection, human immune cells in the ascites of NBSGW-HLA-A2/HHD huPDX3 mice were analyzed by spectral flow and clustered based on a 14-marker human immune panel (Figure 7). Grouping all vaccinated mice together, the percentage of CD86^+^ and CD206^+^ tumor-associated macrophages (TAMs) was significantly lower in the vaccinated groups compared to that of the PBS control group (*p* = 0.0124 and *p* = 0.046, respectively). Conversely, there was a significant increase in the percentage of CD4^+^ T cells in the vaccinated groups (*p* = 0.0129). These data are consistent with repolarization of the tumor microenvironment toward a T helper type 1 (Th1) phenotype following vaccination.

**Figure 8:**
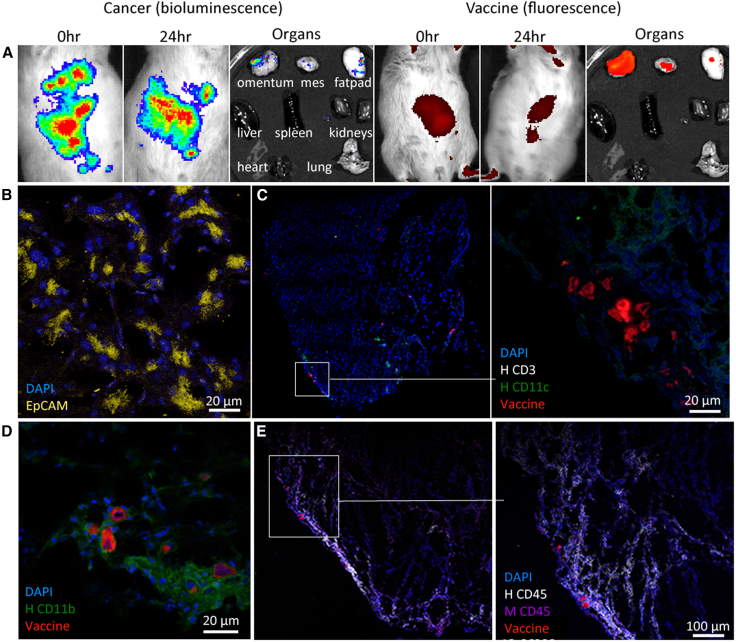
Vaccine biodistribution and cellular associations CTFR vaccine was administered by i.p. injection in a PDX3-Luc2 tumor-bearing female huNBSGW-HLA-A2/HHD mouse 28 days post-tumor engraftment. (A) Tumor (bioluminescence) and vaccine (fluorescence) biodistribution were imaged in live mice and excised peritoneal organs immediately or 24 h after vaccine injection. (B–E) Omental tissue imaging. (B) Anti-human EpCAM (clone 9C4; Alexa Fluor 594; yellow) and DAPI (blue). (C) Anti-human CD11c (clone 3.9; Alexa Fluor 488; green), anti-human CD3 (clone OKT3, Alexa Fluor 647; white), and DAPI (blue) shown at 2 magnifications. (D) Anti-human CD11b (clone M1/70.15; Alexa Fluor 488; green) and DAPI (blue). (E) Anti-human CD45 (clone D9M81; Alexa Fluor 555; white), anti-mouse CD45 (clone D3F8Q, Alexa Fluor 488; purple), and DAPI (blue). Scale bars size representations are stated in each image (20–100 µm).

Vaccine biodistribution and cellular associations were studied in a non-treated, tumor-bearing (28 days post-tumor engraftment) huNBSGW-HLA-A2/HHD mouse. 28 days post-cancer cell injection, CellTrace Far-Red (CTFR) vaccine (3 × 10^6^ cells) was injected i.p. 24 h post-injection, CTFR vaccine cells were located in adipose tissues (54%), predominantly the omentum (71%), and localized to the same tissues as the bioluminescent tumor cells (Figure 8A). Confocal microscopy imaging of fixed omental tissue confirmed the presence of human ovarian cancer cells based on the binding of anti-epithelial cell adhesion molecule (EPCAM) antibody (Figure 8B). Vaccine cells at the periphery of the omentum were not seen associating with CD11c^+^ DCs, with the latter being rare in mice with advanced ovarian cancer (Figure 8C). However, vaccine cells were associated with CD11b^+^ myeloid cells (Figure 8D) and were enriched at sites with human CD45^+^ cells (Figure 8E). At this stage of cancer development, CD3^+^ T cells were sparse in omental sections. Similar to preclinical studies in immune-competent mouse models, the vaccine colocalized with CD11b^+^ myeloid cells and was located predominantly within the adipose tissues at sites rich in immune cells (i.e., milky spots).

## Discussion

Humanized mouse studies began using 4-week-old vendor-bred (the earliest age of delivery) NSG-A2 mice with the human HLA-A2.1 transgene. Immunodeficient mouse strains, such as NSG, are highly permissive for the engraftment of human HSCs based on a lack of innate and adaptive lymphocytes. While these mice are deficient in murine B and T cells, irradiation is necessary for myeloablation. HSC engraftment was via the retro-orbital sinus. This humanized mouse model has the HLA-A2 transgene but not the heavy and light chains that enable better functional maturation of T cells. We noted a decline in humanization over time that may be due to the mouse HSCs outcompeting the human HSCs. However, since we only tested this strain in one experiment, we cannot be certain whether the decline was due to characteristics of the specific donor cells that were implanted or if it is a property of the HLA-A2 strain. While humanization rates were high at 12 weeks post-engraftment, rates fell dramatically within the next 3 weeks. Silicified cancer cell immune therapy led to an initial reduction in tumor growth in select mice. However, this effect waned as humanization rates declined.

In this model, mice, regardless of treatment, displayed an increase in T and myeloid cells over time, with a decline in B cells, as has been reported for humanized NSG mice.^26^ Despite the poor overall anti-tumor response, a large number of vaccinated mice displayed a reduction in PD-1-expressing myeloid cells and an increase in CD8^+^ T cells in the majority of mice (non-significant), suggesting immune activation. There was, however, a significant reduction in CD69 expression by T cells in vaccinated mice, indicating that the remaining T cells were less suppressive, with an increase in their ability to circulate.^27^ While vaccination did not have a significant impact on PD-1 expression in T cells across all mice, elevated expression in three of five mice warrants future studies to ensure long-term anti-tumor activity. Preclinical studies in syngeneic mouse models showed sustained anti-tumor effects with protection from tumor rechallenge.^2^

Moving forward, we initiated in-house breeding of NSG-HLA-A2/HHD mice to facilitate earlier (2 days of age) irradiation and HSC engraftment. In neonates, the liver is the primary hematopoietic site. The HHD designation indicates that mice express HLA class I heavy and light chains, avoiding thymic atrophy and enabling human T cell selection on a human transgene MHC (HLA-A2). Unfortunately, studies using this model resulted in low humanization, with no increases in T and myeloid cells with time.

In previous humanized mouse studies, including our own, the NBSGW mouse strain demonstrated enhanced HSC engraftment with an increase in humanization over time.^19^^,^^28^^,^^29^ HSC engraftment of NBSGW-HLA-A2/HHD mice resulted in superior and sustained humanization rates, with increases in T and myeloid cells over time. Vaccination of mice bearing autologous tumor implants reduced the tumor burden with an underlying increase in T cells and a reduction in TAMs. Pretreatment of vaccinated mice with FLT3-L to encourage DC maturation transiently improved the vaccine response, with a significantly reduced tumor burden at day 20 post-treatment compared to the vaccine group that was not administered FLT3-L. However, the tumor burden increased in 3 mice in the FLT3-L group by day 27, such that there was no significant difference in tumor burden by day 27 between vaccinated mice with or without FLT3-L pretreatment. Based on the initial robust reduction in tumor burden in FLT3-L-treated mice, future studies will determine if sustained FLT3-L dosing renders an enduring impact on vaccine efficacy.

These studies demonstrate that the silicified cancer cell vaccine is able to reduce the ovarian cancer PDX tumor burden in humanized mouse models. The degree of humanization and the host mouse greatly influence the response. An optimal response was seen in huNBSGW-HLA-A2/HHD mice with high humanization. The ovarian cancer immune environment in the ascites fluid of untreated mice shows a high percentage of immunosuppressive myeloid cells and a lower percentage of T cells. Vaccination alters this environment by decreasing the myeloid population and increasing the T cell population. Thus, silicified cancer cell vaccines are able to influence human immune cells and effectively reduce tumor burden in a humanized ovarian cancer PDX model.

i.p. vaccine administration in huNBSGW-HLA-A2/HHD mice with advanced ovarian cancer resulted in the internalization of the vaccine by myeloid cells with trafficking to omental milky spots, similar to that seen in syngeneic mouse models of ovarian cancer.^3^ This study also demonstrated the potential value of targeting transferrin receptor or folate receptors that are overexpressed on ovarian cancer cells for quantification of tumor burden in live mice and at necropsy.

### Conclusions

This study shows the limitations of early humanized mouse models and the importance of developments that advanced the models to be more representative of the human system. Using the NBSGW-HLA-A2/HHD humanized mouse model engrafted with ovarian cancer PDX enabled us to demonstrate that silicified cancer cell vaccines presenting human TLR-targeted CpG ODN 2006 and MPL are effective at reducing human ovarian cancer. Previous studies using preclinical models of ovarian and colon cancer demonstrated robust reductions in tumor burden in vaccinated mice compared to vehicle-treated controls, with T cell-mediated protection from tumor rechallenge.^2^^,^^3^ Studies in humanized mice have enabled us to demonstrate that these outcomes carry over to human systems. i.p. treatment of humanized mice with silicified cancer cell immune therapy mirrored that seen in syngeneic models of ovarian cancer, with the vaccine first trafficked to omental milky spots by myeloid cells, followed by activation of regional immune cells.

## Materials and methods

### Materials

To functionalize silicified cells, CpG ODN 2006 was purchased from InvivoGen (San Diego, CA, USA), MPL from the *Salmonella enterica* serotype was purchased from Sigma (St. Louis, MO, USA), 25K linear polyethylenimine (PEI) was purchased from Polysciences (Warrington, PA, USA), and cell-culture-grade endotoxin-free water was purchased from GE Healthcare (Chicago, IL, USA). Tetramethyl orthosilicate (TMOS), hydrochloric acid bioreagent, sodium chloride (NaCl), and 10% buffered formalin were purchased from Sigma-Aldrich (St. Louis, MO, USA). PBS was purchased from Thermo Fisher Scientific (Waltham, MA, USA), and fetal bovine serum (FBS) was purchased from ATCC (Manassas, VA, USA). 0.05% EDTA trypsin solution and penicillin-streptomycin were purchased from Life Technologies Corporation (Carlsbad, CA, USA). RPMI medium was purchased from Caisson Labs (Smithfield, UT, USA). D-luciferin potassium salt was purchased from PerkinElmer (Boston, MA, USA).

### Methods

#### Cell lines

The PDX (PDX3) cell line was established using malignant ascites obtained during cytoreductive surgery from a patient with ovarian cancer at the University of New Mexico Comprehensive Cancer Center (UNM-CCC) under approved institutional review board protocol INST1509. 20 million ascites cancer cells were injected into the peritoneal cavity of NSG (RRID: IMSR_JAX:005557) mice to create the orthotopic PDX model of disseminated ovarian cancer, as reported previously.^15^ When mice exhibited abdominal extension, ascites fluid was collected by direct insertion of a large-gauge needle into the peritoneal cavity for aspiration. Cells were cryopreserved or serial passaged as needed. Spheroids from PDX3 were lentivirus transduced to constitutively express the bioluminescent reporter Luc2. Transduced spheroids were maintained in CellSTAR cell-repellent microplates (Greiner Bio-One, Monroe, NC, USA) in RPMI containing 20% FBS and 100 units/100 μg penicillin/streptomycin at 37°C and 5% CO.^2^

#### Mouse models

To establish humanized NSG-HLA-A2 mice, 4-week-old female NSG-HLA-A2 (NOD.Cg-Mcph1^Tg(HLA-A2.1)1Enge^Prkdc^scid^Il2rg^tm1Wjl^/SzJ, stock 009617, RRID: IMSR JAX:009617) were purchased from Jackson Laboratories and irradiated with 90 cGy using a MultiRad 225 compact X-ray irradiator, followed by retro-orbital injection of 5 × 10^4^ HLA-matched (HLA-A2.1) cord-blood-derived CD34^+^ HSCs (HumanCells Bio, Milpitas, CA, USA).

NSG-HLA-A2/HHD (NOD.Cg-*Prkdc*^*scid*^*Il2rg*^*tm1Wjl*^Tg(HLA-A/H2-D/B2M)1Dvs/SzJ, RRID: IMSR_JAX:014570), NBSGW-HLA-A2/HHD, and NBSGW (NOD.Cg-*Kit*^*W-41J*^*Tyr*^+^*Prkdc*^*scid*^*Il2rg*^*tm1Wjl*^/ThomJ, RRID: IMSR_JAX:026622) mice were bred in house. All animal protocols were approved by the Institutional Animal Care and Use Committee (IACUC) at the University of New Mexico Health Sciences Center (Albuquerque, NM, USA). Humanized mice were established in 2-day-old NSG-HLA-A2/HHD or NBSGW-HLA-A2/HHD neonates. NSG-HLA-A2/HHD neonates were irradiated with 70 cGy followed by intra-hepatic injection with 4 × 10^4^ HLA-matched (HLA-A2.1) cord-blood-derived CD34^+^ HSCs (HumanCells Bio). NBSGW-HLA-A2/HHD mice did not require pre-engraftment irradiation and were injected with 4 × 10^4^ HLA-matched (HLA-A2.1) cord-blood-derived CD34^+^ HSC (HumanCells Bio) via temporal vein injection. A subgroup of five NBSGW-HLA-A2/HHD mice received 50 μg of premium-grade human FLT-3 ligand protein (Acro Biosystems, Newark, DE, USA) by subcutaneous injection on days 1, 4, and 15 post-tumor challenge.

PDX3-Luc2 cancer spheroids were implanted i.p. to model disseminated ovarian cancer in all humanized mouse models. PDX3 was established by the Steinkamp lab from a patient undergoing cytoreductive surgery at the UNM-CCC.^15^ PDX3 spheroids were transduced with a Luc2 lentivirus (GenTarget) and propagated in NSG mice by the UNM Animal Models Shared Resource. Fresh PDX3-Luc2 spheroids collected from the donor NSG PDX were implanted in humanized mice 14–15 weeks after HSC engraftment by i.p. injection of 5 × 10^6^ cells. huPDX mice were weighed weekly and monitored for signs of distress or wasting.

#### Cryo-silicification of cancer cells

Cancer spheroids obtained from the ascites of an NSG mouse engrafted with PDX3 ovarian cancer were used to create silicified cancer cells. BD Pharm Lyse Lysing Buffer (BD Biosciences) was used to remove red blood cells (RBCs). Cancer spheroids (15 × 10^6^) cells were washed with 10 mL PBS, followed by 10 mL of 154 mM NaCl, and then suspended in 10 mL silicic acid solution containing 10 mM TMOS, 100 mM NaCl, and 1.0 mM HCl (pH 3.0) in a 15 mL conical tube. Following a 10-min incubation at room temperature, the cell suspension was transferred to −80°C for 24 h or longer.

#### Coating silicified cells with cationic polymer and TLR ligand

Silicified cancer cells (15 × 10^6^) were thawed and washed in PBS and then made cationic by suspending the cells in 10 mL of 0.2 mg mL^−1^ 25K linear PEI for 10 min with rotation. After washing with PBS, the supernatant was removed, and 50 μg CpG 2006 (final volume of approximately 100 μL PBS) was added and incubated for 10 min with gentle mixing every 2 min. Next, 25 μg of MPLA was added, and the solution was incubated for another 10 min with gentle mixing every 2 min. The unbound TLR agonist was removed by washing the cells in PBS. The vaccine was resuspended in 1,000 μL PBS.

#### Vaccination of mice

Tumor-bearing mice were vaccinated by i.p. injection with 3 × 10^6^ silicified ascites-derived PDX-3 ovarian cancer cells modified with PEI, MPL, and CpG ODN 2006 in 200 μL of PBS on days 4 and 11. As previously reported, a vaccine dose of 3 × 10^6^ cells contains approximately 5 μg CpG, 4 μg MPL, 54 μg PEI, and 0.2 μg silicon (Si).

#### Imaging tumor burden

For *in vivo* monitoring of tumor burden, mice were administered 150 mg luciferin/kg by i.p. injection, with a 10-min delay prior to imaging. Mice were anesthetized using 2.5% isoflurane, and 2D/3D bioluminescence images were acquired using the IVIS Spectrum *In Vivo* Imaging System (PerkinElmer, Waltham, MA, USA). Region-of-interest (ROI) measurements of total flux (photons/s) or fluorescence (for vaccine) were acquired using Living Image 4.7.3 Software (PerkinElmer). IVISense Transferrin Receptor 750 (excitation [Ex] 745 nm/emission [Em] 800 nm) and Folate Receptor 680 (Ex 680 nm/Em 720 nm) fluorescence imaging agents (PerkinElmer/Revvity) were administered by i.p. or i.v. injection at the recommended dose, followed by longitudinal imaging at specified time points using the IVIS Spectrum configured to acquire images using the corresponding narrow-band Ex/Em filters. Harvested peritoneal organs (omentum, uterus/ovaries, perigonadal fat pads, pancreas, liver, spleen, and kidney) were also imaged on the IVIS to evaluate tumor distribution.

#### Immune cell phenotyping

Peripheral blood was collected from mice between weeks 8 and 15 after HSC engraftment. 100 μL of blood was treated with RBC lysis buffer (Santa Cruz Bio, Dallas, TX, USA), blocked with Human TruStain FCX (BioLegend) in 2.5% fetal bovine serum (FBS)/PBS, and stained with the panel of 5 antibodies (Table S1) at 4°C for 20 min, protected from light. Samples were then washed in 2.5% FBS/PBS and analyzed immediately. Phenotyping was performed using the Attune NxT flow cytometer and analyzed using FlowJo (10.6) (Becton Dickinson, Franklin Lakes, NJ, USA).

#### Spectral flow cytometry

We designed a 14-color antibody panel to immunoprofile major cell subsets from humanized mice: 13 human-specific markers targeting cell surface proteins, 1 mouse-specific CD45 marker, and a Zombie NIR viability stain (Table S2). Fluorophores were distributed across 4 lasers to limit overlap in co-expressing cells. The similarity indices for each fluorochrome were calculated with the Cytek Full Spectrum Viewer (Cytek), and the complexity index was determined to be 7.6.

Ascites fluid was collected from huPDX mice at the endpoint using a 25G needle and syringe, followed by the collection of a 2 mL PBS wash. Cells were centrifuged at 500 × *g* for 10 min at 4°C, and the pelleted cells were treated with ammonium chloride (STEMCELL Technologies) to lyse contaminating RBCs. Cells were washed with serum-free RPMI medium and filtered through cell strainers to remove large aggregates of cancer spheroids and to obtain a single-cell suspension. The remaining cells, consisting of human and mouse immune cells and human cancer cells, were counted on the Countess II (Thermo Fisher Scientific). Cells were pelleted by centrifugation and resuspended in 1 mL of fluorescence-activated cell sorting (FACS) buffer (1% FBS in PBS). Human non-specific immunoglobulin G (IgG) Fc receptors were blocked with 10 μg/mL Human BD Fc Block (BD Pharmingen) for 10 min at room temperature. A portion of cells from each group was set aside to use as unstained controls and as a live-dead reference control. The 14-color antibody cocktail was diluted in FACS buffer supplemented with Brilliant Stain Buffer (BD Horizon) using the final dilutions for each antibody listed in Table S2. Multi-stained cells were stained for 1 h on ice, protected from light. Cells were washed in PBS and stained with the Zombie NIR Fixable Viability Kit (BioLegend) at a 1:10,000 dilution for 15 min at room temperature. Unstained controls were incubated in FACS buffer only. The live-dead single-stain control was prepared with unstained cells that were incubated at 65°C for 10 min to kill cells, followed by staining with Zombie NIR. All samples were washed in FACS buffer and filtered through 5 mL round-bottom tubes with a cell-strainer cap (Falcon). Phenotyping was performed using the Cytek Aurora full-spectrum flow cytometer located in the UNM-CCC Flow Shared Resource. Spectral flow data were unmixed using SpectroFlo Flow Cytometry Software v.3.0 (Cytex Biosciences, Fremont, CA, USA) and analyzed with FlowJo (10.6) (Becton Dickinson). Ultra-Comp eBeads (Invitrogen) were used for single-stain controls, and an autofluorescence signature was designated as an additional fluorescent tag during unmixing. Manual gating removed doublets, debris, and dead cells. Human CD45^+^ immune cells from all samples were further analyzed by t-SNE to cluster immune cell subpopulations.

#### Tissue immunofluorescence

Mice were euthanized in accordance with the IACUC at the University of New Mexico Health Science Center (Albuquerque, NM, USA), and peritoneal organs and tumors were frozen in optimal cutting temperature (OCT) compound. Sectioned tissues were fixed in ice-cold acetone for 15 min, blocked for non-specific binding, and incubated with fluorescent antibodies diluted at 1:50 in PBS with 1% BSA for 1 h at room temperature. Tissues were washed in PBS, and slides were cover slipped using ProLong Gold mounting medium containing DAPI. Images were acquired using a 20× or 63×/1.4 NA oil objective in sequential scanning mode using a Leica TCS SP8 confocal microscope. Tiled images are composites of 975 and 3,295 images taken using a 20× objective and assembled using the Leica stitching algorithm with smooth blending.

#### Statistical analysis

Measurements in this study were obtained from distinct samples. GraphPad Prism 10.0.3 (GraphPad Software, Boston, MA, USA) was used to perform the statistical analysis. For tumor burden comparisons, one-way or two-way ANOVAs with multiple *t* test comparisons were performed, assuming all rows are sampled from populations with the same variability. Where indicated, column statistics were analyzed using unpaired, two-tailed parametric *t* tests with equal standard deviation (SD). Graphs include means and SD error bars.

## Data and code availability

All figures have associated raw data. IVIS images and GraphPad Prism tumor burden data are available as source data. Data are stored in a cloud-based workspace at the University of New Mexico Health Science Center. Datasets are available upon request.

## Acknowledgments

We are grateful for the assistance and use of the University of New Mexico Comprehensive Cancer Center (UNM-CCC) Animal Models, Fluorescence Microscopy, Flow Cytometry, and Human Tissue Repository & Tissue Analysis Shared Resources, supported in part by 10.13039/100000002NIH grant P30 CA118100, and the AIM Center Core, supported by NIH grant P20GM121176. Research was supported by NIH grants R21CA282618 and R01CA293942, the 10.13039/100016438Oxnard Foundation, and the Kleh Family Foundation (principal investigator [PI]: R.E.S.), as well as UNM-CCC pilot project funding (R.E.S. and M.P.S.).

## Author contributions

R.E.S. and M.P.S. wrote the manuscript. R.E.S., M.P.S., D.B., I.L., L.F., and M.M. performed the experiments. R.E.S., M.P.S., D.B., and M.M. analyzed the data.

## Declaration of interests

R.E.S. is an inventor on patents based on silicified cancer cell technology.

## Supplemental information

Document S1. Tables S1 and S2

Document S2. Article plus supplemental information
