# Supplementary material for: Clinical translation of TLR agonist-modified silicified cancer cell therapy supported by humanized mouse models
Source: Mol Ther Oncol. 2025 Oct 24;33(4):201074. doi: 10.1016/j.omton.2025.201074 (PMC12657293; doi:10.1016/j.omton.2025.201074)
Supplement: Document S1. Tables S1 and S2 [file mmc1.pdf]

OMTON, Volume *33*

Supplemental information

Clinical translation of TLR agonist-modified silicified cancer cell therapy supported by humanized mouse models

Mara P. Steinkamp, Danielle Burke, Madigan Morrison, Irina Lagutina, Lillian Fitzpatrick, and Rita E. Serda

Supplemental Data

Table S1: Antibodies used for flow cytometry analysis of blood

Species reactivity	Antibody, supplier	Clone	chromophore
mouse-	CD45 (BioLegend, 103108), 1:2000	30-F11	FITC
human	CD45 (BioLegend, 304012), 1:200	HI30	APC
	CD3 (Life Technologies, 62-0038-42), 1:2000	UCHT1	BV421
	CD11b (Life Technologies, 25-0118-42), 1:300	ICRF44, 44	PECy7
	CD19-PE (BioLegend, 302208), 1:400	HIB19	PE

Table S2: Antibodies used for spectral flow cytometry analysis of immune cells in the tumor micro-environment (purchased from BioLegend)

Marker-fluorochrome	Catalog number	Clone	Dilution
huCD163-BV421	333612	GHI/61	1:200
huCD3-BV605	300460	UCHT1	1:330
huCD4-BV650	300536	RPA-T4	1:1,000
huCD45-BV711	304049	HI30	1:200
huPD-1-BV750	329965	EH12.2H7	1:500
huCD11c-AF488	301617	3.9	1:1,000
muCD45-PerCP	103129	30-F11	1:2,000
huCD66b-PerCP-Cy5.5	305108	G10F5	1:500
hCD80-PE	305207	2D10	1:500
huCD19-PE Dazzle594	302251	HIB19	1:1,000
huCD11b-PE-Cy7	301322	ICRF44	1:500
huCD8-APC	344721	SK1	1:500
hCD206-APC-Fire 750	321133	15-2	1:500
huCD86-647	376304	QA19A61	1:250
